# Transcription Profiles of Marker Genes Predict The Transdifferentiation Relationship between Eight Types of Liver Cell during Rat Liver Regeneration

**DOI:** 10.22074/cellj.2016.3756

**Published:** 2015-07-11

**Authors:** Xiaguang Chen, Cunshuan Xu

**Affiliations:** 1 Animal Science and Technology School, Henan University of Science and Technology, Luoyang, China; 2 Key Laboratory for Cell Differentiation Regulation, Henan Normal University, East of Construction Road, Xinxiang, China; 3 College of Life Science, Henan Normal University, East of Construction Road, Xinxiang, China

**Keywords:** Cell Transdifferentiation, Rat Liver Regeneration, Cell Isolation

## Abstract

**Objective:**

To investigate the transdifferentiation relationship between eight types of liver cell during rat liver regeneration (LR).

**Materials and Methods:**

114 healthy Sprague-Dawley (SD) rats were used in this experimental study. Eight types of liver cell were isolated and purified with percoll density gradient centrifugation and immunomagentic bead methods. Marker genes for eight types of cell were obtained by retrieving the relevant references and databases. Expression changes of markers for each cell of the eight cell types were measured using microarray. The relationships between the expression profiles of marker genes and transdifferentiation among liver cells were analyzed using bioinformatics. Liver cell transdifferentiation was predicted by comparing expression profiles of marker genes in different liver cells.

**Results:**

During LR hepatocytes (HCs) not only express hepatic oval cells (HOC) markers (including *PROM1, KRT14* and *LY6E*), but also express biliary epithelial cell (BEC) markers (including *KRT7* and *KRT19*); BECs express both HOC markers (including *GABRP*, PCNA and THY1) and HC markers such as *CPS1, TAT, KRT8* and *KRT18*; both HC markers (KRT18, KRT8 and WT1) and BEC markers (*KRT7* and *KRT19*) were detected in HOCs. Additionally, some HC markers were also significantly upregulated in hepatic stellate cells ( HSCs), sinusoidal endothelial cells (SECs) , Kupffer cells (KCs) and dendritic cells (DCs), mainly at 6-72 hours post partial hepatectomy (PH).

**Conclusion:**

Our findings indicate that there is a mutual transdifferentiation relationship between HC, BEC and HOC during LR, and a tendency for HSCs, SECs, KCs and DCs to transdifferentiate into HCs.

## Introduction

Mammalian liver is almost unique amongst body tissues in its regenerative capacity (1). The capacity of the liver to regenerate after resection has been known since the late 1800’s (2). From that time onward, numerous scientists have devoted themselves to the study of liver regeneration (LR). However, many questions about LR have not been clearly answered yet, especially with regard to the transdifferentiation activities of different types of liver cell, including hepatocytes (HCs), biliary epithelial cells (BECs), hepatic oval cells (HOCs), hepatic stellate cells (HSCs), sinusoidal endothelial cells (SECs), Kupffer cells (KCs), pit cells (PCs) and dendritic cells (DCs) (3, 4). Of these eight types of liver cell, the latter five cell types are also collectively known as liver non-parenchymal cells. Transdifferentiation means the conversion of one differentiated cell type to another (5). Currently, transdifferentiated cells can be examined using cell function, epigenome, transcriptome, or proteome profiles, or by tracing the expression of markers of the target cell type. Among these methods, measurement of cell specific markers, considered as the potential indicator for identification or tracing the differentiation of specific cell types, is the most utilized approach (6). As for transdifferentiation relationships among different liver cells, at present, many researchers are primarily focused on studying transdifferentation between HOC, HC and BEC and have made significant progress. For instance, many studies have come to the conclusion that HOCs can differentiate into HCs and BECs through the observation that HOC can express markers of both HC and BECs. Briefly, during the course of differentiation of HOC toward HC and BEC, the expression level of HOC markers tends to decrease, while the expression of levels of HC markers (such as *ALB, AFP, G6P, HNF4a, KRT18*) and BEC markers (such as *GGT, KRT7, KRT19*) gradually increase (7-10). An *in vitro* HC culture experiment carried out by Nishikawa et al. (11) showed that, in the course of HC culture, expressions of mature HC markers (such as *ALB, HNF1, HNF4α* and *KRT8*) were gradually lost. In turn, some bile duct-specific proteins (such as *KRT7* and KRT19) began to be expressed, indicating that HCs have the potential to trans-differentiate into BEC. Additionally, transdifferentiation of mature HCs into biliary cells has been shown to occur in rat liver (12). It has also been reported that rat BECs are capable of undergoing hepatic differentiation upon sequential exposure to liver-specific factors. For example, experimental observation of *in vitro* rat BEC culturing by Snykers et al. (13) showed that when rat epithelial cells were exposed to a hepatic-stimulating microenvironment, biliary *KRT19* and connexin CX43 both gradually declined in expression, and *KRT19* expression even disappeared completely. In contrast, expression of HC marker KRT18 persisted throughout the culture process. Furthermore, hepatic *HNFβ, AFP, TTR, HNF4, ALB, HNFα, MRP2* and *CX32* were also strongly expressed, showing the differentiation capacity of BEC into HC. As mentioned above, this research was mainly carried out on the transdifferentiation relationships among HOC, BEC and HC. However, little is known about whether other transdifferentiation activities exist among the eight types of liver cell. For this reason, in this study we separately isolated the eight types of liver cell at 0, 2, 6, 12, 24, 30, 36, 72, 120 and 168 hours after partial hepatectomy (PH) and examined their transcriptional profiles with Rat Genome 230 2.0 Array. We also emphatically analyzed expression changes in the marker genes of the above liver cell types during the regeneration process, and the potential transdifferentiation relationships among these cell types.

## Materials and Methods

### Preparation of rats - the 2/3 hepatectomy model

Animals used in this experimental study are Sprague-Dawley (SD) rats that are obtained from the Animal Center of Henan Normal University. A total of 114 cleaning-grade adult rats, aged 10-12 weeks and weighing 190 ± 20 g were randomly divided into nine PH groups, nine sham-operation (SO) groups and one control group with 6 rats per group. Rats in the PH groups underwent an operation for 2/3 PH according to the guideline described by Higgins and Anderson (14). Briefly, the left and median lateral liver lobes were surgically removed, then the hepatectomized rats were allowed free access to food and water for 2, 6, 12, 24, 30, 36, 72, 120 and 168 hours, respectively, and sacrificed by cervical dislocation. Rats in the SO groups were treated as mentioned above, but no liver lobes were removed. The animals in the control group, as in the case of the 0-hour samples for both the SO and PH groups, were perfused immediately after the surgical removal of left and median lobes. At the same time, the rat body weight (g) and regenerating liver weight (g) were noted and the liver coefficient (L^c^) was calculated using the following formula: L^c^=regenerating liver weight (g)/ body weight (g)×100% (15). All procedures involving rats in this study were performed in accordance with the standard protocols approved by the Ethical Committee of Henan Normal University.

### Isolation of different liver cell types

Rats were subjected to abdominal skin disinfection with alcohol after being anaesthetized by inhaling diethyl ether. The abdominal cavity was opened to expose the liver and the superior and inferior vena cava was ligated followed by portal vein cannulation. The dispersion of liver cells and isolation of different liver cell types were performed according to the method described previously (16). The liver was perfused with calcicum-free perfusate preheated at 37˚C until it turned grey, then with a 15 mL 0.05% collagenase IV solution (Invitrogen, USA) instead of perfusate at a flow rate of 1 mL/minutes. After the liver capsule was removed, the perfused liver was cut into small pieces and digested with 0.05% collagenase IV for 15 minutes at 37˚C. After this it was filtered through 200-well nylon netting (Corning, USA) and the liquid was centrifuged (3S-R low speed refrigerated centrifuge, Leica, Germany) at 500 g for 3 minutes. The pellet at the bottom was collected and washed three times in a 4˚C phosphate buffer saline (PBS) buffer to adjust the cell concentration to 1×10^8^ cells/mL. Six mL of the mixed cell suspension was spread onto the surface of 4 mL 60% percoll (Pharmacia, Biotech AB, Uppsala, Sweden) in a 10 mL tube for a single centrifugation at 200 g for 5 minutes at 4˚C. The centrifuged pellets and supernatant were the purified HCs and nonparenchymal cells-enriched supernatant fractions, respectively. The supernatant was mixed with an equal volume of PBS, centrifuged at 400 g for 2×2 minutes at 4˚C. The mixed nonparenchymal cell-rich pellet was collected and adjusted to a concentration of 1×10^8^ cells/mL, then mixed with 10 μL/mL of rat anti-THY1, -GFAP, -CD31, -CD68, CD161a, and -CD11c PE-antibodies (BD Biosciences, USA), respectively. HOCs, HSCs, sinusoidal endothelial cells, KCs, PCs and DCs were identified using the immunomagnetic bead method (17). White intrahepatic bile duct fractions left on the nylon netting were added into the digestive solution containing 0.25% trypsin (Sichuan Deebio Pharmaceutical Co., Ltd, China) and 0.05% collagenase IV, incubated at 37˚C for 50 minutes, and filtered through 200-well nylon netting. The filtered solution was centrifuged at 300 g for 5 minutes. The resulting sediment was the pellet enriched with BECs. BECs were isolated with rat anti-KRT19 PE-antibody as described above.

### Immunohistochemical identification of eight types of liver cells

A few fractions of each type of liver cell was taken and fixed on glass slides with 10% formaldehyde (Nanjing Senbei Jia Biotechnology Co., Ltd., China) for 30 minutes, then smeared onto glass slides. Microwave antigen retrieval was undertaken once the cell samples on the glass slide had dried. In relation to HCs, for instance, the slides were incubated separately with anti-*ALB* and G6P antibody overnight at 4˚C, then with biotin-labeled secondary antibody at 37˚C for 60 minutes. The reacted sections were mixed with streptavidin-biotin complex (SABC, Wuhan Boster Biological Technology., Ltd., China) and incubated at 37˚C for 30 minutes. Finally, 3,3΄-diaminobenzidine (DAB, Wuhan Boster Biological Technology., Ltd., China) was added for staining and the results observed under an optical microscope (Shang Hai Tuo Feng Instrument Co., Ltd., China) (18). Similarly, BECs, HOCs, HSCs, SECs, KCs, PCs, and DCs were respectively identified with anti-KRT18 and GGT1, OC2 and OV6, CD14 and ET-1, LYZ and ED2, DES and VIM, CD8 and CD56, CD86 and CD103 antibodies following the above protocol.

### Rat Genome 230 2.0 microarray detection and data analysis

Total RNAs from eight types of liver cell at each recovery time point were extracted one by one, according to the Trizol reagent manual (Invitrogen Corporation, Carlsbad, California, USA) and then purified following the RNeasy mini protocol (Qiagen, Inc, Valencia, CA, USA). The quality of the total RNA samples was assessed by agarose electrophoresis (180 V, 0.5 hours) with a 2:1 ratio of 28S rRNA to 18S rRNA intensities and optical density measurement at 260/280 nm prior to cDNA synthesis (19). RNAs pooled from 6 rats in each group were used as a probe. The probes were amplified and biotinylated [Gene Tech (Shanghai) Co., Ltd., China] according to the Affymetrix recommendations for microarray analysis. Probes were hybridized to the Rat Genome 230 2.0 microarray. Arrays were washed to remove the superfluous hybridization buffer, stained in a GeneChip fluidics station 450 (Affymetrix Inc., Santa Clara, CA, USA) and scanned with a GeneChip scanner 3000 (Affymetrix Inc., Santa Clara, CA, USA). The images obtained were converted into normalized signal values, and signal detections [present call (P), absent call (A) and marginal call (M)] values using Affymetrix GCOS 2.0 software (20). To minimize technical error during the array analysis, the Rat Genome 230 2.0 Array procedure was repeated using three liver cell samples at each time point.

The data for each microarray were normalized by scaling all signals to a target intensity of 200 using GCOS 2.0 software (Affymetrix, USA). Each probe set used in the Affymetrix GeneChip produces a detection call, with present expression (P, requiring P value<0.05) indicating good quality, marginal expression (M, requiring 0.05<P value<0.065) indicating intermediate quality and absent expression (A, P value>0.065) indicating relatively low reliability. Therefore, probe sets that resulted in A calls were removed to filter out false positives.

### Identification of differentially expressed genes

Each array was analyzed based on the P, M, or A detection call for probes. The relative values (fold change) in gene expressions were evaluated according to the ratio of the normalized signal value for the surgical groups (including SO groups and PH groups) at different time points to those for the control groups, e.g., genes with a relative value≥3 were regarded as upregulated expression; genes with a relative value≤0.33, as downregulated expression and genes with a relative value between 0.33~2.99 as insignificant expression changes. Relative values of three independent chip analyses at each time point were averaged as effective values.

To look for those genes whose expression changes are truly induced by the LR process, this study compared the fold change in gene expression in the PH groups with that in the SO groups using the F test. In this study, genes showing the same or similar expression trends at the same time points in three independent chip assays, at least a 3-fold change in expression level, and a significant (P≤0.05), or even extremely significant (P≤0.01), difference between the PH groups and SO groups are referred to as genes differentially expressed during LR.

### Quantitative real time polymerase chain reaction (qRT-PCR)

To validate the reliability of the microarray results, RT-PCR analysis was performed. RNA samples were prepared from eight types of liver cell at 10 time points after PH. Primer sequences were designed by primer express 2.0 software, and synthesized by Shanghai GeneCore BioTechnologies Co., Ltd according to the mRNA sequences of eight marker genes *G6PC, GGT1, OC2, GFAP, CD14, LYZ, CD56, CD86* for HC, BEC, HOC, HSC, SEC, KC, PC and DC, respectively (Gen- Bank number: U07993, NM_053840, BG671896, NM_017009, NM_021744, L12458, NM_031521, NM_020081). Total RNA was reverse transcribed with random primers using a reverse transcription kit (Promega, USA). cDNA was amplified using SYBR® Green I on Rotor-Gene 3000 (Corbett Robotics, USA). Standard curve and copy number were evaluated according to the protocol described by Wang and Xu (21).

## Results

### Changes in liver coefficient during rat liver regeneration

**Fig.1:**
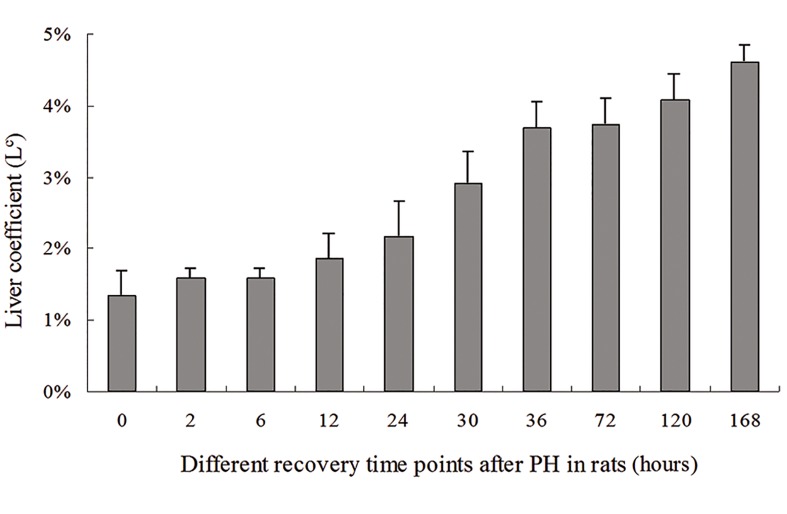
Change in the liver coefficient during rat liver regeneration. PH; Partial hepatectomy.

After the animals were killed at 0, 2, 6, 12, 24, 30, 36, 72, 120 and 168 hours after PH by cervical dislocation, rat body weight (g) and regenerating liver weight (g) were determined and the L^c^ was calculated using the above-mentioned formula. These calculations showed the liver coefficients at the ten different time points post PH were 1.35, 1.58, 1.58, 1.86, 2.17, 2.92, 3.69, 3.74, 4.08 and 4.61% respectively (Table 1) and suggest that the rat liver mass gradually recovered as regeneration progressed (Fig.1).

**Table 1:** Marker-positive rates for eight liver cell types at different time points (hour) after partial hepatectomy in rats


Liver cell types	Markers	Percentage of positive cells at each recovery time (hour) after partial hepatectomy (PH)									
		0	2	6	12	24	30	36	72	120	168

Hepatocyte	*ALB*	98.33	98.06	98.13	97.77	97.65	96.68	96.75	96.43	97.41	97.46
	*G6PC*	97.13	96.46	96.43	96.76	96.33	96.76	96.44	96.89	96.73	96.35
Biliary epithelia cells	*GGT*	95.66	95.31	96.65	95.21	94.78	95.43	96.66	95.55	97.3	95.42
	*KRT18*	96.33	94.41	95.23	97.53	96.21	95.55	96.48	97.04	94.22	96.28
Hepatic oval cells	*OC2*	95.66	95.31	96.65	95.21	95.78	95.43	95.66	95.55	95.3	95.42
	*OV6*	96.33	95.41	95.03	96.53	96.21	95.55	96.48	96.04	95.22	96.28
Hepatic stellate cells	*DES*	96.43	96.5	96.33	96.35	95.43	96.16	96.19	96.55	95.82	96.63
	*VIM*	95.33	95.41	95.03	95.73	95.11	96.55	95.48	96.04	96.22	95.28
Sinusoidal endothelial cells	*ET1*	96.43	96.5	96.33	96.35	95.43	96.16	96.19	96.55	95.82	96.63
	*CD14*	95.33	95.41	95.03	95.73	95.11	96.55	95.48	96.04	96.22	95.28
Kupffer cells	*ED2*	96.66	96.31	96.65	96.21	96.78	96.43	96.66	96.55	96.3	96.42
	*LYZ*	95.33	95.41	97.83	96.53	95.21	95.55	95.48	96.04	95.22	96.28
Pit cells	*CD8*	95.23	95.05	96.24	95.75	95.25	96.18	96.97	96.24	95.51	96.24
	*CD56*	95.02	95.71	95.23	95.21	95.24	96.37	95.75	96.45	96.23	95.44
Dendritic cells	*CD86*	95.23	95.05	96.24	95.75	95.25	96.18	96.97	96.24	95.51	96.24
	*CD103*	95.02	95.71	95.23	95.21	95.24	96.37	95.75	96.45	96.23	95.44


### Validation of the purity of the eight different liver cell fractions

In this study, we employed an immunocytochemistry staining approach to identify eight types of hepatic cells isolated from rat liver regenerating after PH using corresponding specific protein markers, as described previously. Purity of the eight liver cell fractions were statistically analyzed according to the marker-positive cell rate. Results showed that the marker-positive rates at each time point were over 96% for HCs, over 94% for BEC, and over 95% for HOC, HSC, SEC, KC, PC and DC (Table 1), suggesting that the purity of all the liver cell types completely met the requirements for microarray detection.

### Validation of microarray results by quantitative real time polymerase chain reaction (qRT-PCR)

**Fig.2:**
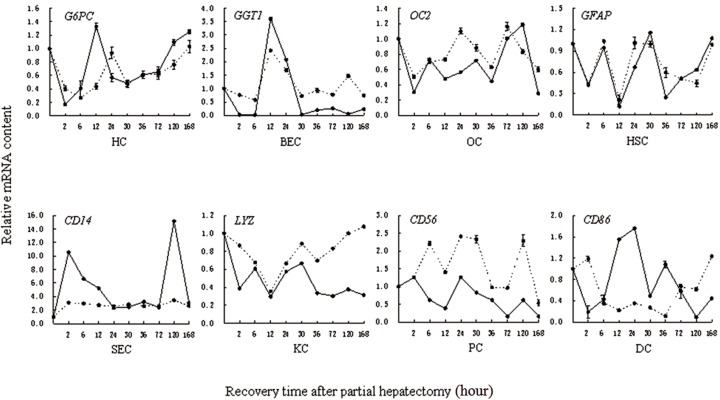
mRNA levels of 8 marker genes for eight liver cell types during rat liver regeneration detected by real time polymerase chain reaction (RT-PCR) and rat genome 230 2.0 array. The results of RT-PCR and microarrays are shown as real lines and broken lines respectively. The recovery time is 2, 6, 12, 24, 30, 36, 72, 120 and 168 hours after partial hepatectomy. HC; Hepatocyte, BEC; Biliary epithelial cell, HOC; hepatic oval cell, HSC; Hepatic stellate cell, SEC; Sinusoidal endothelial cell, KC; Kupffer cell, PC; Pit cell and DC; Dendritic cell.

In this study, quantitative real-time PCR was used to quantify the expression of eight target genes *G6PC, GGT1, OC2, GFAP, CD14, LYZ, CD56* and *CD86* for assessing the reliability of the microarray analysis. As shown in figure 2, the results of both the microarray and quantitative real time (qTR)-PCR analysis indicated insignificant mRNA expressions of *OC2, LYZ* and *CD56*, increased mRNA expression of *GGT1* at 12-30 hours and *CD14* during almost the whole LR, respectively; decreased mRNA expressions of *G6PC* at 24-72 hours, *GFAP* between 2-120 hours after PH, and *CD86* during almost the whole process, except 30 hours. On the whole, based on the data obtained from RT-PCR, with the exception of *LYZ, CD56* and *CD86* whose expression patterns detected by qRT-PCR were not always consistent with those in the array experiments. Expression trends for the other five genes as detected by qRT-PCR were consistent with that detected by microarray, despite the difference in mRNA abundance between the approaches, demonstrating the reliability of the chip results.

### Comparison of the expression profiles of the marker genes for eight types of liver cell during liver regeneration

Through referring to a large number of scientific articles, we found at least 23 HC markers (e.g. *AFP, ALB*), 10 *BEC* markers (e.g. *CD19, KRT19*), 25 HOC markers (e.g. *CD34, c-Met*), 19 HSC markers (e.g. *BDNF, GFAP*), 31 SEC markers (e.g. *CD105, CD11B*), 13 KC markers (e.g. *ACP5, CD14*), 13 PC markers (e.g. *CD161A, CD8A*) and 41 DC markers (e.g. *ADAM19, BDCA2*). Correspondingly, there were 15, 4, 15, 13, 12, 5, 6 and 12 markers (a total of 79 genes) present on the rat genome 230 2.0 array. Based on the stringent standards described in the "Materials and Methods" section, out of the above 79 genes, 14, 18, 13, 25, 20, 23, 11 and 33 markers were significantly differently expressed in the above eight types of liver cell, respectively. As shown in table 2, which shows the maximal or minimal fold-change values for mRNA expression of the genes detected by array, the upregulated genes in three types of liver parenchymal cells were predominant in number, and a majority of genes in the other five nonparenchymal cells were downregulated in expression. However, there had a large difference in the differentially-expressed genes between different types of liver cell. Briefly, HC mainly up-expressed marker genes for BEC and HOC, BEC mainly expressed marker genes for HC and HOC, and HOC up-expressed marker genes for HC and BEC. Meanwhile, four other types of liver cell, including HSC, SEC, KC and DC, individually down-regulated a majority of the marker genes for the other seven types of liver cell.

**Table 2:** Expressions of marker genes in eight different liver cell types


Gene name for markers	Gene symbol	Expression trends of marker genes in 8 different liver cell types							
		HC	BEC	HOC	HSC	SEC	KC	PC	DC

1. HC marker genes									
		*alpha-fetoprotein*	*Afp*					-4.80	3.79		
	*albumin*	*Alb*		-79.69					-6.42	-3.43
	*carbamoyl-phosphate synthetase 1*	*Cps1*		25.27		-10.59	22.30		-17.04	7.48/-12.5
	*glucose-6-phosphatase, catalytic*	*G6pc*			-4.32		7.43		-6.08	3.27/-5.0
	*hepatocyte growth factor*	*Hgf*	18.65							
	*Hepatocyte nuclear factor 4 alpha*	*Hnf4a*								
	*keratin 18*	*Krt18*	4.84	4.54	3.88	3.28/-9.09	13.92	5.88	-3.31	7.19
	*keratin 8*	*Krt8*	3.15	5.30	3.48	-9.48	4.76	4.94		9.62
	*cytosolic phosphoenolpyruvate carboxykinase 1*	*Pck1*		-9.78		-3.78	5.95		-5.88	
	*serine (or cysteine) proteinase inhibitor A1*	*Serpina1*		-7.34		-7.11			-10.85	-8.90
	*tyrosine aminotransferase*	*Tat*		32.44		10.20/-7.69	22.54	-4.61	-17.59	5.14
	*Transferrin receptor protein 2*	Trfr2		-33.62		19.27/-3.85	14.72		-5.88	-3.55
2. BEC marker genes									
		*Gamma-glutamyltransferase 1*	*Ggt1*								
	*tryptophan 2,3-dioxygenase*	*Krt1*								
	*keratin 19*	*Krt19*	7.59		4.40	-7.29	5.32			-5.56
	*keratin 7*	*Krt7*	22.69		39.86	-5.04		5.82		
3. HOC marker genes									
		*cadherin 22*	*Cdh22*								
	*claudin 7*	*Cldn7*								
	*gamma-aminobutyric acid (GABA-A) receptor, pi*	*Gabrp*		14.29						
	*glypican 3*	*Gpc3*					11.98			
	*kit ligand*	*Kitl*								
	*keratin 14*	*Krt14*	13.71						6.33	7.99
	*Lymphocyte antigen 6 complex, locus E*	*Ly6e*	4.21			-7.87		-4.58		-9.59
	*mucin 1, transmembrane*	*Muc1*								
	*proliferating cell nuclear antigen*	*Pcna*		5.49		-3.88				
	*prominin 1*	*Prom1*	8.20		6.85			7.33		5.07
	*PTK2 protein tyrosine kinase 2 beta*	*Ptk2b*						-4.08		
	*protein tyrosine phosphatase, receptor type, C*	*Ptprc*				-7.45	-6.40	-8.06		-17.17
	*Ros1 proto-oncogene*	*Ros1*								
	*SCO cytochrome oxidase deficient homolog 1*	*Sco1*								
	*thymus cell antigen 1, theta*	*Thy1*		5.18						
4. HSC marker genes									
		*smooth muscle alpha-actin*	*Acta2*								
	*brain derived neurotrophic factor*	*Bdnf*			6.45			19.69		
	*Desmin*	*Des*								
	*glial fibrillary acidic protein*	*Gfap*								
	*nerve growth factor*	*Ngf*								
	*nerve growth factor receptor*	*Ngfr*		10.60	8.69		4.32			6.35
	*neurotrophin 3*	*Ntf3*	4.28			21.88		3.96		
	*neurotrophin 5*	*Ntf5*								
	*Neurotrophic tyrosine kinase, receptor, type 2*	*Ntrk2*								
	*neurotrophic tyrosine kinase, receptor, type 3*	*Ntrk3*								
	*platelet derived growth factor receptor, beta polypeptide*	*Pdgfrb*								8.99
	*synaptophysin*	*Syp*								
	*vimentin*	*Vim*	8.61	-4.38		-8.96				-7.87
5. SEC marker genes									
		*alanyl (membrane) aminopeptidase*	*Anpep*								
	*CD14 molecule*	*Cd14*	-5.24	5.07		-2.05				-4.57
	*CD4 antigen*	*Cd4*			-4.05			-6.68		-9.14
	*CD44 antigen*	*Cd44*						-4.57	5.21	
	*endothelin 1*	*Edn1*								
	*Fc fragment of IgG, low affinity IIa, receptor (CD32)*	*Fcgr2a*		5.01		-6.24				-12.26
	*Fc fragment of IgG, low affinity IIIa, receptor*	*Fcgr3a*				-12.26		-5.21		-12.26
	*kinase insert domain protein receptor*	*Kdr*	-8.09	-4.61	-6.85	-24.07	-8.56			-34.65
	*low density lipoprotein receptor*	*Ldlr*								
	*mannose receptor, C type 1*	*Mrc1*		5.84		-9.61				-15.16
	*platelet/endothelial cell adhesion molecule 1*	*Pecam1*				-7.01		3.38		-5.94
	*Von Willebrand factor homolog*	*Vwf*	17.86	4.06	4.03					
6. KC marker genes									
		*acid phosphatase 5, tartrate resistant*	*Acp5*				-5.78				-5.26
	*CD68 antigen*	*Cd68*		3.39/-20		-9.09		-15.51		-18.87
	*glucose-6-phosphate dehydrogenase X-linked*	*G6pdx*				-4.52				-7.46
	*gap junction protein, beta 6*	*Gjb6*	15.53						18.93	13.26
	*lysozyme 2*	*Lyz2*					-6.28			-5.26
	6. KC marker genes									
		*acid phosphatase 5, tartrate resistant*	*Acp5*			-5.78					-5.26
	*CD68 antigen*	*Cd68*		3.39/-20	-9.09		-15.51			-18.87
	*glucose-6-phosphate dehydrogenase X-linked*	*G6pdx*			-4.52					-7.46
	*gap junction protein, beta 6*	*Gjb6*	15.53						18.93	13.26
	*lysozyme 2*	*Lyz2*				-6.28				-5.26
7. PC marker genes									
		*cell cycle related kinase*	*Ccrk*								
	*CD8 antigen, alpha chain*	*Cd8a*				-8.32	-11.49	-15.51		-6.76
	*coenzyme Q10 homolog A (yeast)*	*Coq10a*								
	*interleukin 2 receptor, alpha chain*	*Il2ra*								-9.41
	*killer cell lectin-like receptor subfamily B, member 1A*	*Klrb1a*				-5.52	-5.82	-14.88		
	*neural cell adhesion molecule 1*	*Ncam1*								
	8. DC marker genes									
*Cd2 molecule*		*Cd2*					-4.75	-19.39		
	*CD40 molecule, TNF receptor superfamily member 5*	*Cd40*					-11.30			-8.09
	*CD80 antigen*	*Cd80*								
	*CD83 antigen*	*Cd83*			-4.41		-4.36			-10.13
	*cd86 antigen*	*Cd86*				-4.52		-3.99		-8.79
	*interleukin 3 receptor, alpha chain*	*Il3ra*								
	*integrin, alpha D*	*Itgad*						-15.30		
	*integrin, alpha E, epithelial-associated*	*Itgae*								
	*integrin alpha L*	*Itgal*				-9.26	-4.71	-5.83		-11.93
	*integrin beta 2*	*Itgb2*				-10.21				-8.02
	*kyphoscoliosis peptidase*	*Ky*								
	*S100 calcium binding protein A1*	*S100a1*			8.18			6.23		


Positive and negative values denote the maximum-fold upregulation and downregulation compared with control samples, respectively. Blank boxes represent the insignificant expression of genes. HC; Hepatocytes, BEC; Biliary epithelial cells, HOC; Hepatic oval cell, HSC; Hepatic stellate cell, SEC; Sinusoidal endothelial cell, KC; Kupffer cell, PC; Pit cell and DC; Dendritic cell.

### Cell transdifferentiation reflected by the transcription profiles of marker genes of eight types of rat liver cell during liver regeneration

**Fig.3:**
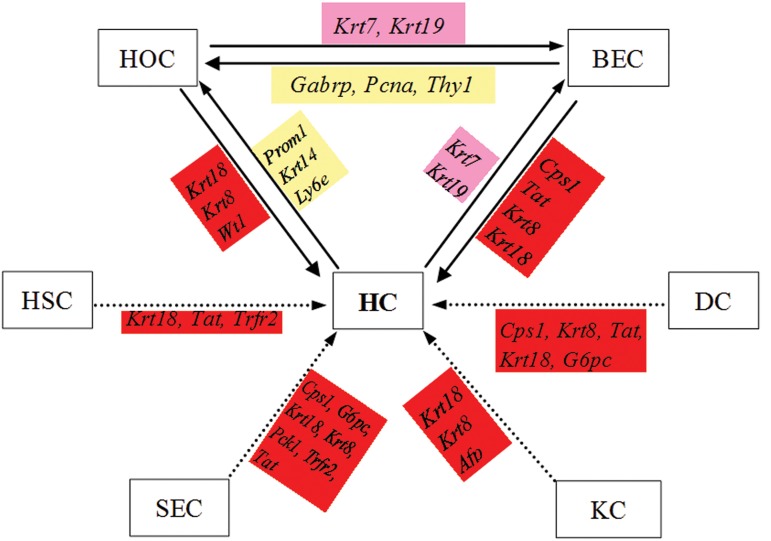
Schematic diagram indicating the transdifferentiation relationships between different liver cell populations during rat liver regeneration reflected by the expression of marker genes for eight liver cell types. Solid arrows denote the reported cell transdifferentiation relationship; Dash-line arrows denote the cell transdifferentiation relationships predicted by this study.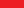; Hepatocyte marker genes,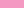 ; Biliary epithelia cell marker genes,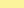 ; Hepatic oval cell marker genes, HOC; Hepatic oval cell, BEC; Biliary epithelial cell, DC; Dendritic cell, KC; Kupffer cell, SEC; Sinusoidal endothelial cell, HSC; Hepatic stellate cell and HC; Hepatocyte.

Based on the transcript abundance of the marker genes for 8 types of liver cell during rat LR (Table 3) HCs expressed dual markers of HOC (*PROM1, KRT14* and *LY6E*) and BEC (*KRT7* and *KRT19*) at 12-72 hours post-PH. After 12 hours biliary lineages began to express oval cell markers *GABRP, PCNA* and *THY1*, and up to 30 hours there appeared a remarkable increase in mRNA levels of HC markers (including *CPS1, TAT, KRT8, KRT18*) in BECs. According to the transcriptional profiles of oval cells, the expressions of marker genes for HCs (*KRT18* and *KRT8*) and that for BECs (*KRT7* and *KRT19*) were detected at 6-36 hours and at 2-168 hours post-surgery, respectively. The observations indicate that HC, HOC, and BEC have, at least limited, multi-differentiation potentials during rat LR. In addition, some markers for HCs were detected in another four liver cell types at 6-36 hours post-surgery, including HC makers *KRT18, TAT, TRFR2* in HSC, HC makers *CPS1, G6PC, KRT18, KRT8, PCK1, TAT* and *TRFR2* in SEC, and HC makers *KRT18, KRT8* and *AFP* in KC, and HC makers CPS1, G6PC, KRT18, KRT8 and Tat in DC, which might give some signs of differentiation of these liver cells towards HCs (Fig.3).

**Table 3:** Transcription profiles of gene markers of 8 types of liver cells in hepatocyte during rat liver regeneration.


Cell types	Marker gene	Recovery times (hour) after rat 2/3 hepatectomy (PH)									
		0	2	6	12	24	30	36	72	120	168

HC	Afp	1.00	0.43	0.36	0.30	24	0.66	0.47	0.53	120	120
	*Alb*	1.00	0.43	0.78	1.20	0.64	1.81	0.95	0.51	0.81	0.81
	*Cps1*	1.00	0.43	0.37	0.37	1.30	1.11	1.17	0.69	1.06	1.06
	*G6pc*	1.00	0.43	0.65	0.29	0.41	0.35	0.61	0.54	0.75	0.75
	*Hgf*	1.00	0.43	1.23	2.73	2.76	1.91	2.54	1.65	1.43	1.43
	*Hnf4a*	1.00	0.43	0.81	0.86	1.52	1.29	1.96	2.53	1.19	1.19
	*Krt18*	1.00	0.43	0.27	2.24	2.29	1.15	2.68	1.71	1.84	1.84
	*Krt8*	1.00	0.43	0.87	1.15	1.52	2.54	2.73	1.28	1.57	1.57
	*Pck1*	1.00	0.43	1.01	1.28	0.83	1.34	0.82	0.85	1.03	1.03
	*Serpina1*	1.00	0.43	0.85	0.86	1.25	0.84	1.63	2.37	1.10	1.10
	*Tat*	1.00	0.43	1.45	1.16	1.14	1.35	1.37	1.33	1.04	1.04
	*Trfr2*	1.00	0.43	1.10	1.04	1.03	1.05	1.51	1.94	1.17	1.17
BEC	Ggt1	1.00	0.43	1.13	1.39	1.74	1.34	1.39	1.51	1.34	1.34
	*Krt1*	1.00	0.43	1.25	1.77	2.61	2.45	2.56	3.12	2.15	2.15
	*Krt19*	1.00	0.43	1.31	1.34	1.73	0.99	2.19	7.59	1.01	1.01
	*Krt7*	1.00	0.43	2.71	3.14	3.82	1.46	3.30	22.69	1.24	1.24
OC	Cdh22	1.00								86	0.86
	*Cldn7*	1.00	0.43	1.48	1.06	1.38	1.49	1.02	1.67	1.01	1.01
	*Gabrp*	1.00	0.43	1.77	1.43	1.74	2.05	1.25	1.46	1.23	1.23
	*Gpc3*	1.00	0.43	2.58	1.22	3.40	1.58	1.42	6.53	1.16	1.16
	*Kitl*	1.00	0.43	0.79	1.05	0.45	0.78	0.40	0.47	0.58	0.58
	*Krt14*	1.00	0.43	2.31	3.17	1.19	1.10	2.39	13.71	2.83	2.83
	*Ly6e*	1.00	0.43	0.96	0.82	1.19	1.70	2.23	4.21	2.11	2.11
	*Muc1*	1.00	0.43	1.04	1.11	1.28	1.09	1.41	1.32	1.04	1.04
	*Pcna*	1.00	0.43	1.57	0.77	1.82	1.30	1.27	7.17	2.08	2.08
	*Prom1*	1.00	0.43	0.66	1.63	1.70	4.18	8.20	1.63	1.31	1.31
	*Ptk2b*	1.00	0.43	2.24	1.57	2.15	1.62	2.02	2.61	1.52	1.52
	*Ptprc*	1.00	0.43	2.09	3.48	5.85	2.74	2.14	2.77	2.72	2.72
	*Ros1*	1.00	0.43	0.67	0.72	0.86	0.86	1.13	0.99	0.91	0.91
	*Sco1*	1.00	0.43	1.61	0.75	2.76	2.43	1.41	1.60	1.47	1.47
	*Thy1*	1.00	0.43	1.89	2.80	5.21	7.83	5.23	3.70	3.07	3.07
HSC	Acta2	1.00	0.88	0.93	1.08	1.59	0.87	1.13	1.60	1.03	0.83
	*Bdnf*	1.00	1.49	1.63	3.53	1.72	1.29	3.01	12.55	4.72	2.72
	*Des*	1.00	1.25	0.31	0.31	0.67	1.80	1.60	1.58	2.35	1.46
	*Gfap*	1.00	0.83	1.29	0.75	2.24	2.74	2.13	2.34	2.12	1.83
	*Ngf*	1.00	1.23	2.91	2.34	2.33	2.22	2.00	2.13	1.85	2.40
	*Ngfr*	1.00	1.38	1.72	2.06	1.13	2.25	1.53	2.61	1.67	1.02
	*Ntf3*	1.00	1.34	1.92	2.60	2.05	0.55	1.09	1.79	1.80	2.84
	*Ntf5*	1.00	1.12	1.11	2.90	1.09	1.90	0.96	1.20	2.69	1.02
	*Ntrk2*	1.00	1.27	1.21	1.46	1.23	1.24	0.96	1.55	1.24	1.05
	*Ntrk3*	1.00	1.02	0.84	1.01	1.27	0.76	1.20	1.70	1.61	1.12
	*Pdgfrb*	1.00	1.15	1.36	0.96	1.65	2.04	1.74	2.00	1.70	1.50
	*Syp*	1.00	1.53	1.79	2.08	1.17	1.63	1.31	2.44	1.94	1.34
	*Vim*	1.00	1.24	1.08	1.33	1.80	3.16	2.16	8.61	2.21	1.79
SEC	Anpep	1.00	1.81	2.36	1.48	1.85	0.99	1.75	2.90	1.57	1.45
	*Cd14*	1.00	1.10	1.53	0.94	0.19	0.35	0.19	0.38	0.71	0.89
	*Cd4*	1.00	0.69	0.71	0.80	0.60	0.80	0.84	0.82	0.83	1.48
	*Cd44*	1.00	1.12	0.79	0.86	2.61	2.53	2.96	2.76	0.88	1.57
	*Edn1*	1.00	2.03	1.58	1.57	1.97	2.30	2.03	1.28	1.41	1.86
	*Fcgr2a*	1.00	2.92	1.34	1.05	1.27	1.94	1.63	1.30	1.30	1.65
	*Fcgr3a*	1.00	0.77	0.87	1.03	0.96	1.48	1.19	1.21	1.02	0.88
	*Kdr*	1.00	0.22	0.33	0.12	1.18	0.77	1.16	5.35	2.33	1.57
	*Ldlr*	1.00	0.97	0.70	0.70	0.45	0.31	0.46	0.44	0.48	0.50
	*Mrc1*	1.00	0.42	0.75	0.70	1.26	1.66	1.63	2.30	2.08	1.18
	*Pecam1*	1.00	1.14	5.04	1.48	2.39	3.21	1.84	4.40	0.98	1.13
	*Vwf*	1.00	2.34	1.04	1.93	6.41	1.90	1.28	17.86	4.28	6.42
KC	Acp5	1.00	1.50	1.43	1.09	0.89	1.03	0.79	0.87	1.25	1.11
	*Cd68*	1.00	1.12	2.07	1.91	1.97	1.22	1.67	1.53	1.36	1.55
	*G6pdx*	1.00	1.16	1.19	0.73	0.79	1.35	1.24	1.48	1.32	1.31
	*Gjb6*	1.00	1.48	6.40	4.09	6.89	5.16	15.53	12.07	5.26	8.43
	*Lyz2*	1.00	2.55	2.44	2.49	1.64	2.30	1.51	0.94	0.99	1.14
PC	Ccrk	1.00	1.38	2.31	2.38	1.63	1.62	1.44	1.61	1.33	1.28
	*Cd8a*	1.00	2.22	2.65	1.18	2.34	2.03	2.94	2.20	2.03	2.04
	*Coq10a*	1.00	0.67	0.28	0.45	0.57	1.17	1.01	0.80	0.90	1.20
	*Il2ra*	1.00	0.86	1.26	2.59	1.44	1.45	1.80	1.42	1.66	1.58
	*Klrb1a*	1.00	0.84	1.01	1.25	1.57	1.13	1.39	1.25	1.10	0.74
	*Ncam1*	1.00	0.69	1.09	1.27	0.83	1.00	1.66	1.75	0.75	2.41
DC	Cd2	1.00	1.40	1.45	1.23	1.68	2.38	2.13	2.43	2.94	2.67
	*Cd40*	1.00	1.11	1.76	2.06	1.63	2.09	2.08	2.72	1.90	1.28
	*Cd80*	1.00	1.10	1.65	0.97	0.91	1.36	0.91	1.44	1.09	1.40
	*Cd83*	1.00	0.94	1.18	1.05	1.52	0.93	1.48	1.32	1.30	1.34
	*Cd86*	1.00	1.10	1.34	1.43	1.39	2.14	1.70	1.11	1.04	1.02
	*Il3ra*	1.00	0.85	1.83	2.01	0.94	1.29	1.34	1.31	0.82	2.46
	*Itgad*	1.00	0.56	0.83	1.69	1.50	1.73	1.65	1.12	0.52	1.23
	*Itgae*	1.00	1.68	1.04	1.06	1.42	0.70	1.32	1.18	0.95	0.79
	*Itgal*	1.00	0.89	0.96	1.11	1.30	0.95	1.20	1.21	1.43	1.18
	*Itgb2*	1.00	1.01	1.01	1.37	1.12	1.02	1.05	0.83	1.14	1.29
	*Ky*	1.00	1.83	1.38	1.57	1.59	1.99	0.69	1.95	1.49	1.16
	*S100a1*	1.00	0.96	0.69	1.14	0.97	1.50	1.23	1.03	1.06	1.11


HC; Hepatocytes, BEC; Biliary epithelial cells, OC; Oval cell, HSC; Hepatic stellate cell, SEC; Sinusoidal endothelial cell, KC; Kupffer cell, PC; Pit cell, DC; Dendritic cell and PH; Partial hepatectomy. The red-colored bins represent ≥3-fold up-regulation, the green-colored bins represent ≥3-fold down-regulation and colorless bins represent insignificant expression.

### Biological activities associated with transdifferentiation of eight types of liver cell during rat liver regeneration

**Fig.4:**
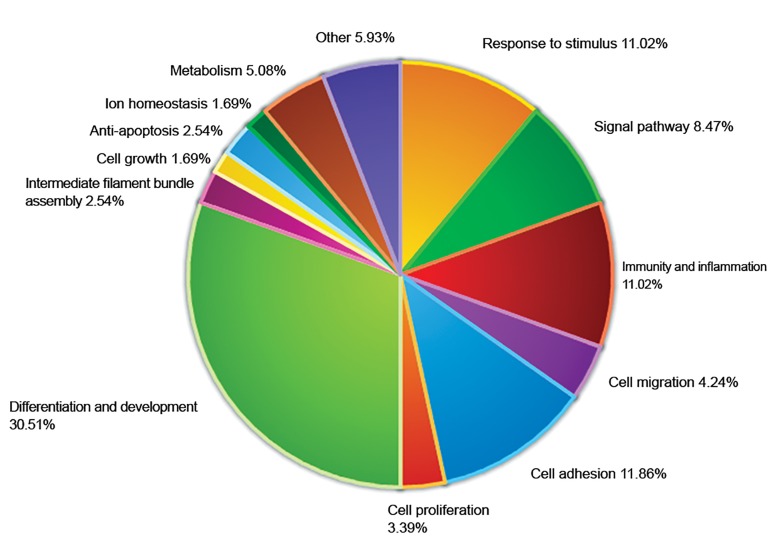
Biological activities involved in trans-differentiation of eight liver cell types during rat liver regeneration. The Pie chart represents the number of genes in each functional group.

Although the above genes serve as the markers for corresponding liver cells, each has its own special mission. Using the gene ontology (GO) category method, we analyzed the biological functions and processes of these 79 significantly expressed markers among 8 liver cell types during LR. The result showed that the above 79 genes were involved in many biological activities, such as response to stimuli, signaling pathways, immunity and inflammation, cell migration and adhesion, differentiation and development, cell proliferation, cellular metabolism, and so on. Contrastingly, amongst them, cell proliferation, differentiation, development, and apoptosis were the predominant biological processes. Notably, genes involved in responses to stimuli, signaling pathways, immunity and inflammation, cell adhesion, and differentiation and development included the largest proportion of genes (11.97, 8.55, 11.11, 11.11, and 30.77%), respectively (Fig.4). Genes involved in important biological activities (i.e., response to stimuli, signaling pathways, immunity and inflammation etc) are listed in table 4.

**Table 4:** Genes involved in response to stimuli, signaling pathways, immunity and inflammation, cell adhesion, and differentiation and development


Process	Genes

Response to stimuli	*Alb, Cd14,Cd83, Cd86, Cps1, Edn1, Fcgr3a, Krt19, Ldlr, Lyz2, Pcna, Ptk2b, Serpina1*
Signaling pathways	*Cd8a, Fcgr2a, Il2ra, Il3ra, Kdr, Ncam1, Ngfr, Pdgfrb, Ptk2b, Syp*
Immunity and inflammation	*Acp5, Bdnf, Cd14, Cd40, Cd44, Cd68, Cd86, Cd8a, Fcgr3a, Il2ra, Krt1, Ngf, Ptprc*
Cell adhesion	*Cd2, Cd4, Cd44, Cdh22, Cldn7, Itgad, Itgae, Itgal Itgb2, Kitl, Ncam1, Ptk2b, Thy1, Vwf*
Differentiation & development	*Acp5, Acta2, Afp, Anpep, Bdnf, Cd4, Cd83, Cd86, Cd8a, Cdh22, Edn1, Fcgr2a, Gfap, Gpc3, Hnf4a, Kdr, Krt14, Krt18, Krt19, Krt7, Krt8, Ky, Ly6e, Muc1, Ngf, Ngfr, Ntf3, Ntf5, Ntrk2, Ntrk3, Prom1, Ptk2b, Ptprc, Ros1, S100a1, Vim*


## Discussion

To explore transdifferentation relationships among different types of liver cell during LR, we isolated eight types of cell with a high degree of purity and vitality at 10 different time points after PH. We then detected the transcriptional profiles of the eight types of liver cell, laying special emphasis on analyzing expression changes in the marker genes for each liver cell type. Results showed that many marker genes for specific liver cell types were expressed in other liver cell types during rat LR. For instance, at 12-72 hour PH, during which time HCs undergo active protein expression and cell division, HCs expressed dual markers for HOC (*PROM1, KRT14, LY6E*) and BEC (*KRT7, KRT19*). According to studies by others, the three HOC markers have the effect of inducing cell differentiation (22-24), while the two BEC markers are specifically expressed during BEC differentiation (25). Meanwhile, marker genes for HCs showed reduced (*AFP, KRT18*, etc.) or insignificant (*Hnf4a, TAT,* etc.) expression. Based on the above analysis, it can be inferred that HC have the potential to differentiate toward HOC and BEC, consistent with Nishikawa’s report of transdifferentiation of HC to BEC (11). From 12 hours after PH, three HOC markers (*PCNA, THY1, GABRP*) and four HC markers (*CPS1, KRT18, KRT8, TAT*) were strongly expressed in BEC cells whose own markers were not significantly expressed or were even lower than in the control group. GO analysis showed that *PCNA* contributes to cell cycle progress through promoting DNA replication (26); *THY1*, acting as specific gene for HOC, is implicated in the formation of stem cells (27). Up-expressions of these genes are a sign of BEC transformation toward HOC. Of four the HC markers, KRT18 and KRT8 specifically promote HC differentiation through modulating actin organization (28), and *TAT* and *CPS1* are involved in amino acid metabolic activity occurring in HC (29). However, contrary to our findings, Snykers et al. (13) found that the HC markers upregulated in BECs were *AFP* and *ALB*, instead of *KRT18, KRT8, TAT* and *CPS1*. This discrepancy may be attributed to differences between our method and Snykers’ method. At 30-36 hours, expressions of HC markers *KRT18, KRT8* and BEC markers *KRT7, KRT19* were detected in HOC. As stated previously, the keratin family can promote cell differentiation (25), suggesting that oval cells are inclined to differentiate into HC and BEC, reconfirming the conclusion that HOCs can act as precursor cells for both HC and BEC (30). According to the above-mentioned data, it suggests that HC, HOC and BEC have multi- differentiation potentials during LR.

In addition, some HC markers were significantly expressed in other liver non-parenchymal cells during LR. At 2-72 hours PH, only the HC markers (*KRT18, TAT, TRFR2*) were obviously upregulated in HSC whose own marker genes showed no significant changes in mRNA levels, suggesting the tendency for HSC transdifferentiation towards HC. Somewhat differently, the results of the study by Chen et al. (10) indicated that HSC induce differentiation of oval cells into mature HCs, instead of directly differentiating into HCs. Accordingly, this might be the first study to report that HSC transdifferentiate into HC. Over the same period, seven HC markers (*KRT18, KRT8, G6PC, CPS1, PCK1, TAT, TRFR2*) were upregulated in SEC whose own markers did not obviously change. In addition, between 6-36 hours KCs and DCs predominantly expressed three (*KRT18, KRT8* and *AFP*) and six specific genes (*CPS1, G6PC, KRT18, KRT8, TAT*) for HC, respectively. As described above, these genes either accelerate cell differentiation or embody the biological functions specific to HCs replicate to restore the original size and function of the liver after PH in rats. Of eight liver cell types, HCs make up 70-80% of the liver mass and 65% of the total cell number. Therefore, recovery of the HC number is of primary importance after PH. While it is well-known that a majority of HCs enter into cell proliferation to compensate for the lost HCs immediately after PH, our analysis suggests that the compensatory HC production may originate partly from the transdifferentation of non-parenchymal cells. This may be the first report on the transdifferentiation relationship between non-parenchymal cells and HCs.

## Conclusion

The observation that markers for any cell type of HOC, HC and BEC are expressed in the other two types of liver cell suggests the potential mutual transdifferentiation among the three types of liver cell during LR. Additionally, four non-parenchymal cells (HSC, SEC, KC and DC) were detected to strongly express HC markers, indicating some signs of differentiation in these four liver cells towards HCs. However, genechip can only test the transcriptional profiles of genes; it is not able to reflect the more exact or direct transdifferentiation activities of different liver cells. In future, we will further test these transdifferentiation relationship using methods such as immunohistochemistry, RNA Interference etc.
