# Transcription Profiles of Marker Genes Predict The Transdifferentiation Relationship between Eight Types of Liver Cell during Rat Liver Regeneration

**DOI:** 10.22074/cellj.2015.21

**Published:** 2015-10-07

**Authors:** Xiaoguang Chen, Cunshuan Xu

**Affiliations:** 1 Animal Science and Technology School, Henan University of Science and Technology, Luoyang, China; 2 Key Laboratory for Cell Differentiation Regulation, Henan Normal University, East of Construction Road, Xinxiang, China; 3 College of Life Science, Henan Normal University, East of Construction Road, Xinxiang, China

In this article which was published in Cell J, Vol 17, No 2, Summer 2015, on pages 339-354, the name of the first author was published incorrectly as: "Xiaguang Chen". The correct one is "Xiaoguang Chen".

This correction was requested by the Corresponding author

